# Anti-PF4 ELISA-Negative, SRA-Positive Heparin-Induced Thrombocytopenia

**DOI:** 10.3390/hematolrep16020029

**Published:** 2024-05-09

**Authors:** Abraham Attah, Chelsea Peterson, Max Jacobs, Rama Bhagavatula, Deep Shah, Robert Kaplan, Yazan Samhouri

**Affiliations:** 1 Department of Internal Medicine, Allegheny Health Network, Pittsburgh, PA 15212, USA; 2 Division of Hematology and Cellular Therapy, Allegheny Health Network Cancer Institute, Pittsburgh, PA 15212, USAyazan.samhouri@ahn.org (Y.S.)

**Keywords:** anti-PF4, serotonin-release assay, heparin-induced thrombocytopenia, venous thromboembolism

## Abstract

Heparin products are frequently used in the inpatient setting to prevent and treat venous thromboembolism, but they simultaneously put patients at risk of developing heparin-induced thrombocytopenia (HIT). The 4Ts score determines the pretest probability of HIT. Diagnosis is made with a screening antiplatelet factor (PF4) immunoassay and the serotonin-release assay (SRA) as a confirmatory test. Anti-PF4 assays have high sensitivity (98%) but lower specificity (50%) and result in frequent false-positive tests. We present a rare case from our institution of a patient with anti-PF4–Polyanion ELISA-negative, SRA-positive HIT and describe the challenges in making a timely diagnosis in this case.

## 1. Objectives

- Briefly describe heparin-induced thrombocytopenia (HIT), diagnosis, and management;
- Describe a very rare case of anti-PF4 ELISA-negative, SRA-positive HIT;
- Highlight alternate antigens that could explain anti-PF4 ELISA-negative, SRA-positive HIT.

## 2. Background

Heparin formulations are frequently utilized in the inpatient setting for the prophylaxis of venous thromboembolism (VTE) and for treating VTE and acute coronary syndrome. Heparin-based products have many advantages in this setting, including a short half-life, which allows for an interruption of procedures, relatively wide accessibility, and reasonable cost. However, the major disadvantage of heparin use is the risk of developing heparin-induced thrombocytopenia (HIT). HIT is an immune-mediated reaction due to antibody formation against the platelet factor 4(PF4)/heparin complex. This complication is relatively rare, occurring in up to five percent of patients receiving unfractionated heparin (UFH). However, it can be fatal in up to 10% of cases [1,2].

Per the guidelines of the American Society of Hematology (ASH), calculating the 4T score is initially recommended whenever HIT is suspected [3,4]. If the score shows intermediate or high pretest probability (≥4), the recommended test is an anti-PF4/heparin complex enzyme-linked immunosorbent assay (ELISA), henceforth referred to as an anti-PF4 assay. This test is very sensitive with a low false-negative rate [5]. Therefore, a negative test would rule out HIT. But given its variable specificity, a positive test should be confirmed by a platelet function assay/serotonin-release assay (SRA), which is highly specific [6]. Here we describe a challenging case of negative anti-PF4 assay but positive SRA with clinical characteristics of HIT.

## 3. Case Presentation

**Figure 1:**
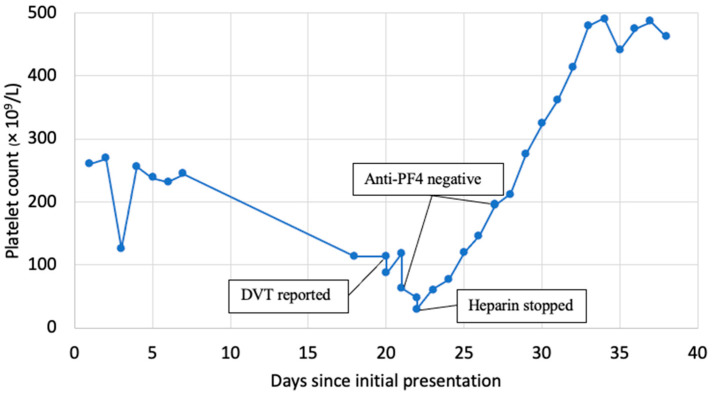
The trend of platelet count and key events.

A 74-year-old female with a history of Parkinson’s disease, chronic obstructive pulmonary disease, and spinal stenosis presented from home for altered mental status. Her mental status changes were thought to be secondary to polypharmacy, her home medications were adjusted, and she was discharged to an inpatient rehabilitation facility. Of note, she was started on subcutaneous unfractionated heparin 5000 units every 8 h for deep vein thrombosis (DVT) prophylaxis during hospitalization, which was continued at the rehab facility. Her platelets at the original admission were 261 × 10^9^/L and 224 × 10^9^/Lon when transferred to rehab. Sixteen days after her initial heparin dose, the patient developed left lower extremity swelling and pain while in rehab. A venous Doppler ultrasound revealed extensive occlusive acute DVT from the left common femoral vein through her popliteal vein. Notably, the platelets dropped to 80 × 10^9^/L on the day DVT was diagnosed (Figure 1). 

She was started on an intravenous unfractionated heparin infusion titrated to a partial thromboplastin time of 70–95 s. She developed worsening pain and swelling of the extremity within hours with the loss of pulses and a further drop in her platelets to 63 × 10^9^/L. A left lower extremity angiogram was obtained, which showed extensive DVT involving the entire left lower extremity venous system with a concern for phlegmasia cerulea dolens. There was a high suspicion of HIT then, so a 4T score was calculated. The patient scored 2 points for a platelet count drop of >50% with nadir >20, 1 point for onset after day 10, 2 points for new thrombosis with apparent acute worsening with intravenous heparin, and 1+ point for possible other causes, including consumptive thrombocytopenia from her extensive DVT. This cumulated to 6 points, indicating a high probability (64%) of HIT. Following this, anti-PF4 assay testing (Asserachrom IgG assay-Stago, Parsippany, NJ, USA) and SRA (Labcorp tests 150014, 150016, and 150017, Burlington, NC, USA) were performed, the heparin drip was stopped, and the patient was started on intravenous argatroban. She was taken for emergent mechanical thrombectomy and subsequent fasciotomy for compartment syndrome while maintained on argatroban.

The anti-PF4 assay returned at 0.21 optical density (OD) (Normal ≤ 0.3999 OD); thus, argatroban was discontinued, and intravenous unfractionated heparin was restarted. After resuming heparin, the patient developed worsening lower extremity pain, and platelets further decreased to 31 × 10^9^/L (nadir). She also developed chest pain and was found to have a non-ST elevation myocardial infarction (NSTEMI) on an electrocardiogram. A left heart catheterization revealed evidence of triple vessel disease. Given her comorbidities and ongoing medical complexities, the patient was managed medically. She was transitioned back to argatroban due to high clinical suspicion for HIT despite a negative anti-PF4 assay, and platelets increased to 76 within 48 h. A serotonin-release assay (SRA) using low-dose unfractionated heparin (0.2 IU/mL) showed an 85% release as well as a <1% release using high-dose unfractionated heparin (1000 IU/mL). This is a positive result as there was a release of >20% in low-dose heparin and inhibition of release in high-dose heparin. These labs were repeated given the high concern for lab error, revealing a normal anti-PF4 assay of 0.154 OD with the SRA being positive at 115% release using low-dose heparin and 14% release using high-dose heparin. Unfortunately, the patient did not have viable tissue of the left lower extremity following these interventions and required an above-the-knee amputation of the left leg. Platelet improved to >200 within a week. She was eventually bridged from argatroban to Warfarin.

## 4. Discussion

HIT is a rare but potentially fatal immune-mediated reaction to heparin characterized by antibody formation to the heparin/PF4 complex, resulting in uncontrolled platelet activation and thus clotting in the presence of heparin. Our case of a patient with a negative anti-PF4 assay but positive SRA is only the second documented case of its kind that we know of [7].

The anti-PF4 assay has a high sensitivity of >99%, with a negative predictive value (NPV) of 98–99%. It does, however, have a low specificity of about 50%. Over-detection of anti-PF4/heparin antibodies leads to a low positive predictive value (PPV) of 10–50%. In contrast to the anti-PF4 assay, the SRA has a higher specificity of >95% with a positive predictive value of 89–100% but a lower and variable sensitivity of 56–100% with a negative predictive value of 81% [2]. The existence of HIT despite a negative anti-PF4 assay poses a therapeutic dilemma when the clinical concern for HIT is high and SRA is pending. Per the 2018 ASH guidelines for the management of VTE in HIT, in patients with a high probability 4T score but a negative immunoassay, it is recommended to discontinue nonheparin anticoagulant and resume heparin if anticoagulation is indicated given the high sensitivity of the anti-PF4 assay in ruling out HIT [4].

In a review By Warkentin et al., 8546 cases were isolated in the same laboratory in Wisconsin. Of these cases, only 0.2% (16 patients) had a negative anti-PF4 assay and positive SRA. These cases were individually analyzed, and it was found that in all cases, there were laboratory or clerical errors to account for the false-negative anti-PF4 assay results [7]. One possible explanation for this patient’s diagnostic workup is the potential of a different target antigen being responsible for the immune reaction. Other antigens implicated in the pathogenesis of HIT include chemokines such as IL-8, protamine, and NAP-2. These have been found in <1% of patients being worked up for HIT and are typically heparin-independent [8,9,10]. Interestingly, studies looking at platelet activation triggered by antibodies to IL-8 have found that although platelet activation and thrombosis occur independently of heparin, adding heparin does increase the platelet response to antibodies in some cases [11,12].

False-negative anti-PF4 assays are rarely documented and typically due to lab errors [7]. There was consideration for lab error in our case, but repeat testing confirmed that the initial testing results were accurate. Additionally, after investigation, another patient had a positive anti-PF4 assay on the day of this patient’s initial negative anti-PF4 assay test, making lab error less likely. To help solidify our report, we had considered running samples against other ELISA assays, including LIFECODES IgG/A/M, but given that the patient had passed, it was difficult to obtain consent from family to proceed with this testing.

## 5. Conclusions

Our case adds to the sparse literature on the clinical conundrum of HIT testing discordance. This case either identifies limitations of the anti-PF4/SRA diagnostic approach or identifies a phenotype of HIT that differs from the norm. Furthermore, it highlights the importance of interpreting HIT testing in the proper clinical context as resuming heparin in these cases can be associated with serious and avoidable life- or organ-threatening complications. Clinicians and patients would benefit from large-scale studies looking further into the concept of HIT testing discordance.

## Data Availability

Data is available upon request.

## References

[1] Arepally G.M. (2017). Heparin-induced thrombocytopenia. Blood.

[2] Salter B.S., Weiner M.M., Trinh M.A., Heller J., Evans A.S., Adams D.H., Fischer G.W. (2016). Heparin-Induced Thrombocytopenia: A Comprehensive Clinical Review. J. Am. Coll. Cardiol..

[3] Lo G.K., Juhl D., Warkentin T.E., Sigouin C.S., Eichler P., Greinacher A. (2006). Evaluation of pretest clinical score (4 T’s) for the diagnosis of heparin-induced thrombocytopenia in two clinical settings. J. Thromb. Haemost..

[4] Cuker A., Arepally G.M., Chong B.H., Cines D.B., Greinacher A., Gruel Y., Linkins L.A., Rodner S.B., Selleng S., Warkentin T.E. (2018). American Society of Hematology 2018 guidelines for management of venous thromboembolism: Heparin-induced thrombocytopenia. Blood Adv..

[5] Sun L., Gimotty P.A., Lakshmanan S., Cuker A. (2016). Diagnostic accuracy of rapid immunoassays for heparin-induced thrombocytopenia. A systematic review and meta-analysis. Thromb. Haemost..

[6] Marchetti M., Barelli S., Zermatten M.G., Monnin-Respen F., Matthey-Guirao E., Nicolas N., Gomez F., Goodyer M., Gerschheimer C., Alberio L. (2020). Rapid and Accurate Bayesian Diagnosis of Heparin-induced thrombocytopenia. Blood.

[7] Warkentin T.E., Smythe M.A., Ali M.A., Aslam N., Sheppard J.I., Smith J.W., Moore J.C., Arnold D.M., Nazy I. (2021). Serotonin-release assay-positive but platelet factor 4-dependent enzyme-immunoassay negative: HIT or not HIT?. Am. J. Hematol..

[8] Tardy B., Lecompte T., Mullier F., Vayne C., Pouplard C. (2020). Detection of Platelet-Activating Antibodies Associated with Heparin-Induced Thrombocytopenia. J. Clin. Med..

[9] Strobel E. (2017). Use of the ID-PaGIA Heparin/PF4 Antibody Test as a screening test for heparin/platelet factor 4 antibodies. Blood Transfus..

[10] Castelli R., Cassinerio E., Cappellini M.D., Porro F., Graziadei G., Fabris F. (2007). Heparin Induced Thrombocytopenia: Pathogenetic, Clinical, Diagnostic and Therapeutic Aspects. Cardiovasc. Hematol. Disord. Targets.

[11] Regnault V., de Maistre E., Carteaux J.-P., Gruel Y., Nguyen P., Tardy B., Lecompte T. (2003). Platelet activation induced by human antibodies to interleukin-8. Blood.

[12] Warner M., Pavord S., Moore J., Warkentin T., Hayward C., Kelton J. (1999). Serum-induced platelet procoagulant activity: An assay for the characterization of prothrombotic disorders. J. Lab. Clin. Med..

